# Probing the Microstructure in Pure Al & Cu Melts: Theory Meets Experiment

**DOI:** 10.3389/fchem.2020.00607

**Published:** 2020-08-07

**Authors:** Lin Song, Xuelei Tian, Yanmei Yang, Jingyu Qin, Hui Li, Xiaohang Lin

**Affiliations:** ^1^Key Laboratory for Liquid-Solid Structural Evolution and Processing of Materials, Ministry of Education, Shandong University, Jinan, China; ^2^Key Laboratory of Molecular and Nano Probes, Ministry of Education, Collaborative Innovation Center of Functionalized Probes for Chemical Imaging in Universities of Shandong, College of Chemistry, Chemical Engineering and Materials Science, Institute of Molecular and Nano Science, Shandong Normal University, Jinan, China

**Keywords:** liquid structures, Al & Cu metallic melts, wulff shape, high-temperature XRD, DFT

## Abstract

In the present work, a new model of the atomic cluster structure, which is determined by metal Wulff construction with the crystal structure inside, is proposed to describe the structures of metallic melts. The shapes of the structures are determined by surface energies of different crystal plane groups, calculated from density functional theory (DFT), while the size is given by the pair distribution function (PDF) of the experimental high-temperature X-ray diffraction (HTXRD). Taking Aluminum (Al) and Copper (Cu) as the representative examples, we demonstrate that the simulated XRD curves from present models match the experimental results quite well, not only regarding the position and width of the peaks but also the relative intensity of the first and second peaks. These results indicate a successful model to describe the properties of metallic melts. The model also explains a main peak deviation phenomenon between the XRD of metallic melt and the solid ones in pure metal Al. Finally, a physical picture of metallic melt is given, which is mainly composed of atomic cluster structures and free atoms around them.

## Introduction

Around the world, higher requirements have been put forward for metallic material use in areas such as energy conservation, environment protection, etc. (Zhang et al., 2014a,b). Higher strength, hardness, lighter weight, and other superior physical and chemical properties (like electrochemistry, catalysis) are commonly required (Alayoglu et al., 2008; Ahmad and Singh, 2015; Zheng et al., 2017). A deeper understanding of the structures of metal melts are essential for the design and production of many metallic materials. For example, both the solidification processes of casting metals and the glass transition processes of amorphous alloys begin with the metallic melts (Kita et al., 1994; Debenedetti Pablo and Stillinger, 2001; Ganesh and Widom, 2008; Pan et al., 2015). In other words, melts are the “parent states” of metallic materials. The microstructure and physical/chemical properties of metallic materials are determined by the compositions and structures of their melts. As a result, it is necessary to understand metallic melts clearly.

In recent years, it has been reported that high-temperature X-ray diffraction (HTXRD), synchrotron X-ray diffraction (XRD), and X-ray absorption fine-structure (EXAFS) are common methods used to observe the metallic melts directly (Turnbull and Nagel, 1990; Li and Pederiva, 2003; Li et al., 2003; Xue et al., 2003; Lou et al., 2013). The short-range ordering and distribution of the internal structure in melts can be estimated by calculating the structure factor and the pair distribution function (PDF) (Li and Pederiva, 2003; Li et al., 2003). However, due to the high-temperature and liquid condition in the experiment, the basic physical images of the structures are covered by complex appearances and have remained unclear.

To form a relatively simple model is a common way to deal with such a complicated system, which could show the basic physical images directly and clearly. Numerous approximate models have been proposed to explain the phenomena observed by experiments, such as the crystal defect model, quasi-crystalline model, and atomic clusters model (Bernal, 1959; Susskind and Becker, 1966; Schenk et al., 2002; Huang et al., 2011). It is widely accepted that metallic melts are composed of atomic clusters (Lou et al., 2013; Turnbull and Nagel, 1990; Bernal, 1959; Susskind and Becker, 1966; Li and Pederiva, 2003; Li et al., 2003; Xue et al., 2003; Huang et al., 2011; Schenk et al., 2002; Itami et al., 2003; Jakse and Pasturel, 2003; Shintani and Tanaka, 2006; Sreeja Kumari et al., 2007; Hwang et al., 2008; Hou et al., 2009; Seifeddine and Svensson, 2010; Vasisht et al., 2011; Sha et al., 2012; Li and Li, 2014; Zhai et al., 2014). According to this model, the metallic melts are composed of a large number of atomic clusters (short-range ordered) in the environment of free atoms (disordered), which is consistent with the results of the PDF in HTXRD. Unfortunately, it is impossible for existing models to describe some experimental phenomena. For example, there is a main peak deviation phenomenon between the XRD of metallic melts and the solid ones in pure metal Al (Tian et al., 2010). Although there are reports about the study of the atomic clusters model using the molecular dynamics (MD) methods (Jakse and Pasturel, 2003; Li and Pederiva, 2003; Li et al., 2003; Vasisht et al., 2011), the thermodynamic equilibrium structure and morphology of clusters in melts have not been studied at the atomic level.

For the thermodynamic equilibrium system, Wulff theory is suitable to describe the structure of nanoparticles (Tyson and Miller, 1977; Tran et al., 2016). According to the theory, the structure and morphology of nanoparticles in crystals are determined by the surface energies of planes on the condition of thermodynamic equilibrium (Gilman, 1960). Wulff theory relates the polar plot of a given material's anisotropic surface energies, which is the shape with the lowest surface energy for a given volume, and can easily be determined in the following way. First, radius vectors must be drawn from the origin of the polar plot of the surface energies. Then, at the points of the intersections, a plane perpendicular to the corresponding radius vector must be constructed, and then these planes should be connected with the lowest surface energy to form a closed convex shape.

In our paper, due to the particularity of the HTXRD results in Al melt, we decided to study Al and selected the common casting metal Cu as the contrast. The surface energy of the crystal facet can be obtained by the first principle method based on density functional theory (DFT). Moreover, the Wulff shape was given to construct a stable crystal structure in a thermodynamically stable metallic melt. Compared with the results of experiment HTXRD, this model describes the structures of atomic clusters in various pure metals (including Al and Cu) well. At the same time, the main peak deviation between the liquid HTXRD results and the solid ones is also explained by our model. This general physical model is provided for the description of pure metallic melts, and the extension of the models to binary alloys is also in progress.

## Methods

### Experimental Methods

The pure Al and Cu ingots (both 99.999%) were employed for liquid X-ray diffraction experiments in this work. The experiments were carried out using θ-θ high-temperature X-ray diffractometer. Mo Kα radiation (wavelength λ = 0.07089 nm) is reflected from the free surface of the liquid specimen and reaches the detector through a graphite monochromator in the diffraction beam. HTXRD was carried out in a high purity helium (99.999%) atmosphere (1.3 × 10^5^ Pa) before the chamber was cleaned in a vacuum (2 × 10^−6^ Pa). The samples were placed in an aluminum oxide crucible with a size of 30 × 25 × 8 mm, which was heated by a Ta sheet. They were overheated to 1,500°C, held for 1 h, and then cooled down to the measurement temperature. The surface of the specimen was fitted to one horizontal position using a laser calibrator. The X-ray parameters were set as follows: scanning voltage was 40 kV, current was 30 mA, exposure time was 30 s, and the measured angle (2θ) was from 5 to 80°.

The PDF describes the distribution of other atoms around one, which reflects the correlation of atomic density in a multiparticle system. The PDF is usually applied to analyze XRD results of crystal, amorphous structures, and melt to obtain structure and size parameters, which is defined as followed.

(1) $$\begin{array}{r} \text{g}\left(\text{r}\right)=\frac{\Omega \langle n_{i}\left(r,r+\Delta r\right)\rangle }{4\pi r^{2}\Delta rN} \end{array}$$

where 〈*n*_*i*_(*r, r*+Δ*r*)〉 is the average number of atoms between *r* and *r*+Δ*r*, *N* is the total number of atoms in a certain system, and Ω is the volume of a unit cell. The PDF describes the probability of other particles appearing around a characteristic particle. The experimental X-Ray diffraction intensity was converted to structure factor *S(Q)* after a polarization, absorption correction, and normalization procedure. The pair distribution function *g(r)* is obtained by the Fourier transform of the structure factor *S(Q)*, and the conversion formula is as follows:

(2) $$\begin{array}{r} g\left(r\right)=1+\frac{1}{2\pi r^{2}\rho_{0}}\overset{\infty }{\underset{0}{\int }}Q\left[S\left(Q\right)-1\right]sinQrdQ \end{array}$$

Where $Q=\frac{4\pi sin\theta }{\lambda }$ and ρ_0_ is the number density of the metal at certain temperatures.

### Theoretical Models

First-principles calculations, based on periodic density functional theory, are known to yield a satisfactory description of crystal structures and alloy properties (Hohenberg and Kohn, 1964; Kohn and Sham, 1965). All the simulations in this paper are performed by the Vienna ab initio simulation package (VASP) (Dulub et al., 2005; Lin et al., 2014) within the generalized gradient approximation (GGA) to describe the exchange-correlation effects, using the Perdew, Burke and Ernzerhof (PBE) exchange-correlation functional which gives a good description of metallic surface properties (Perdew et al., 1996). After the convergence test was carried out precisely, the energy cut-off value was set as 400 eV for plane wave expansions in reciprocal space. Energy calculations were performed in the first irreducible Brillouin zone using 15 × 15 × 1 k-point by the Monk horst-Pack scheme to confirm a good convergence of total energy (Monkhorst and Pack, 1976). The surface structures are fully relaxed to ensure that the atomic and lattice parameters are in the most stable energy state. The structures were relaxed until the residual forces were smaller than 0.015 eV/Å and the value of energy convergence accuracy was 1.0 × 10^−5^ eV/atom. To evaluate the accuracy of the models, benchmark calculations of Al and Cu crystals were first conducted using the above setup parameters. Our calculations gave lattice constants of 2.855 and 2.568 Ã for Al and Cu, respectively, both in the FCC structure, showing good agreements with previous studies (Suh and Waseda, 1988; Tougait and Noël, 2004).

To accurately evaluate the formation energies of different crystal surfaces, the typical slab model was used in the calculations. This model is constructed by selectively exposing the plane of interest and removing a portion of atoms to form a vacuum. All slab models are constrained to the symmetrical top and bottom surfaces. We determine the pure metal's surface energy γ using the slab model given by the following equation:

(3) $$\begin{array}{r} \gamma =\frac{1}{2A}\left(E_{slab}-NE_{bulk}\right) \end{array}$$

where *A* is the total area of the facet, *E*_*slab*_ means the generated energy of the generated slab model, *E*_*bulk*_ is the energy of the bulk unit cell, and N is the number of atoms in bulk structure.

The cluster structures are all relaxed when using implicit solvent DFT calculations. The outermost two layers are fully relaxed. To calculate the diffraction pattern, the software *Materials Studio* (Rietveld refinement, Rietveld with energies, Pareto optimization, and modified Pawley refinement based Rietveld, 1969; Pawley, 1981; Engel et al., 1999; Van Veldhuinzen and Lamont, 2000) was used. In order to consider the temperature effect, the diffraction curves should be broadened. The broadening peak profile is defined as (Post, 1974; Cullity, 1978; Kern et al., 2004; Tian et al., 2010):

(4) $$\begin{array}{l} I(2\theta )=\sum_{i=1}^{n}\{I_{i}[\frac{P_{1}}{\sqrt{a_{1}^{2}+b^{2}}\sqrt{2\pi }}e^{-\frac{{\left(2\theta -2\theta_{i}\right)}^{2}}{2\left(a_{1}^{2}+b^{2}\right)}}+\\ \frac{P_{2}}{\sqrt{a_{2}^{2}+b^{2}}\sqrt{2\pi }}e^{-\frac{{\left(2\theta -2\theta_{i}\right)}^{2}}{2\left(a_{2}^{2}+b^{2}\right)}}]\}\;+G[1-\;D^{2}(s)] \end{array}$$

where *I*_*i*_ represents the intensity of the number *i* XRD peak of a crystal lattice, *P*_1_ is the ratio of the amount of the atoms in the inner part of the atomic short-range ordering, *P*_2_ is the ratio of the amount of the atoms in the surface part, *a*_1_ and *a*_2_ are coefficients that indicate the *I*(2θ) breath, and *b* is a coefficient that is related to the breath of the broadening peak. 2θ is the XRD angle and 2θ_*i*_ is the position of the peak *i* of a crystal lattice. The *D*^2^(*s*) = *e*^−*B*^*s*^^2^/2^, B means a temperature coefficient present during the effect of the thermal vibration and *s* = 2sinθ/λ, G is the coefficient of the background.

## Results and Discussion

For one sample, the liquid XRD experiments gave exactly the same results, which indicates two important points. First, the metallic melts are in the condition of thermodynamic equilibrium and the structural distribution is kept constant. Second, the peaks of the experimental results show that it has short-range ordering in metallic melts. In this case, the thermodynamic equilibrium condition allows us to use Wulff construction theory, which is proven to be a good model to describe the equilibrium shape of the crystal (Gilman, 1960; Tyson and Miller, 1977; Tran et al., 2016). Wulff construction can easily determine the shape of the atomic cluster, but what about the structures inside? Comparing the XRD results of solid and metallic melt, though the positions of peaks does not directly match, especially for Al (mentioned in details below), it is still obvious that the XRD curves of metallic melt are more or less related to the solid ones. Hence, an assumption that the internal structure of atomic clusters should exhibit the characteristics of the crystal structure was made. This bold hypothesis is partially proven by the evolution of optical absorption spectra of gold atomic clusters with various sizes (Tian et al., 2015; Lee et al., 2016). According to the previous studies (Gilman, 1960; Tyson and Miller, 1977; Tran et al., 2016), a new model of the cluster structure is proposed to describe the structure of metallic melts, which is determined by metal Wulff construction with the crystal structure inside.

### Experimental Results

**Figure 1:**
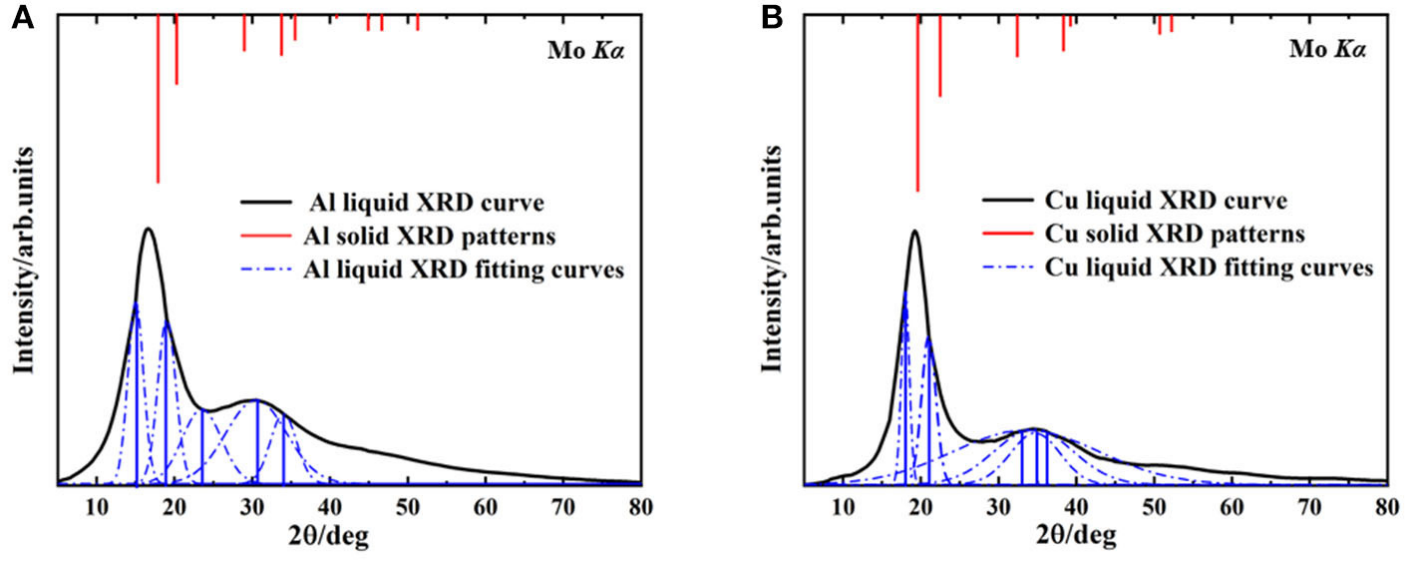
The liquid XRD curves and solid XRD patterns, **(A)** Aluminum at 1,250°C, **(B)** Copper at 1,350°C.

The XRD spectrums of Al melt at 1,250°C, Cu melt at 1,350°C, and XRD intensity of the solid are shown in Figure 1. The black curves are the XRD of metallic melt samples measured by HTXRD. The red lines represent the XRD intensity of solid samples and the blue lines are the fitting XRD intensity of liquid ones. There are two reasons for choosing the specific temperature. First, it's convenient to compare with the previous report to verify the accuracy of the results (Tian et al., 2010). Second, due to the limitation of computing resources, we prefer to simulate the smaller atomic clusters at a higher temperature rather than larger ones at a lower temperature.

In Al and Cu melts (Figure 1), the first peaks are both (111) crystal plane group, and their second peaks correspond to (100) crystal plane group. Compared with the solid ones, diffraction peaks of liquid curves are much broader, which is mainly caused by the temperature effects. Note that a deviation of the main peak in the Al melt happened obviously (from 17.6 to 15°, the deviation reached 14.7%) and the ratio of relative intensity about the first and second peaks were also changed significantly (from about 2.5:1 in solid to 10:9 in melt), shown in Figure 1A. As a contrast, in the Cu melt (Figure 1B), there was smaller deviation (from 19.6 to 18.1°, the deviation was 7.6%) and no significant change of relative strength between the first and second peaks. These results are consistent with the previous reports (Tian et al., 2010) and can hardly be explained by present metallic melts models. Our following investigation shows that this phenomenon is determined by the specific cluster structure in melts.

**Figure 2:**
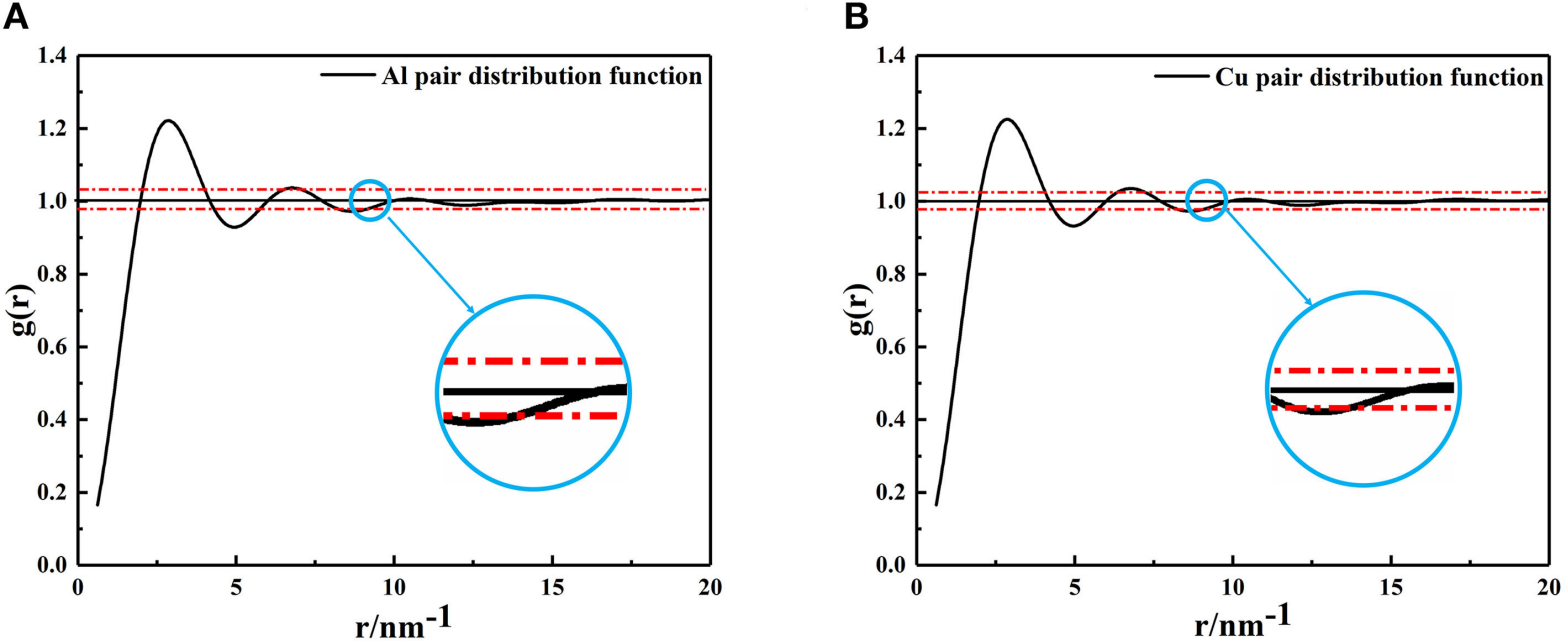
The pair distribution function curves, **(A)** Aluminum at 1,250°C, **(B)** Copper at 1,350°C. The last intersection position of red dashed lines and PDF curves were enlarged, and the abscissa corresponding to the intersection is the radius of the cluster.

Using the PDF (Equation 1), the average size of nano-particles in the metallic melts were calculated. Generally speaking, the value of formula *g(r)* = 1 ± 0.02 is usually defined as a short-range ordered range and atomic correlation is also thought to disappear beyond this range (Turnbull and Nagel, 1990; Kaiser et al., 2002; Li and Pederiva, 2003; Li et al., 2003; Xue et al., 2003). The XRD results of metallic melts were taken into equation (1) to form the PDF shown in Figure 2. As mentioned above, when the amplitude of *g(r)* is <0.02, the correlation between particles disappears. It is not hard to find that the last intersection shown in a blue circle (Figure 2) represents the size of the metallic melt at that temperature. The radius of Al and Cu clusters are, respectively, determined to be 9.158Ã (number of atoms: 192) and 9.198 Ã(number of atoms: 274).

### Theoretical Model Results

**Figure 3:**
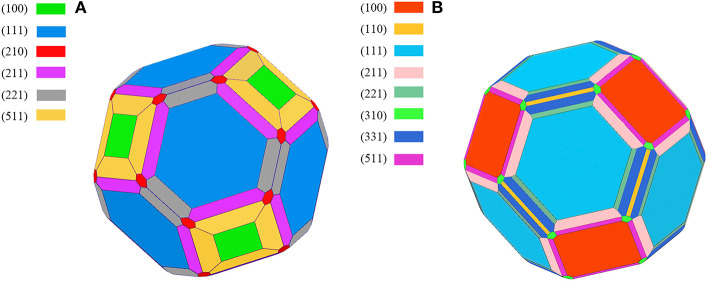
The Wulff shape of pure metal, **(A)** Aluminum, **(B)** Copper, various surfaces are shown by different colors.

The calculated results of different crystal planes of two metals are shown in Table 1. For the low-index surfaces of the Al and Cu, trend γ(111) < γ(100) < γ(110) of surface energy is obvious, and the highest results of Al and Cu are, respectively, γ(310) and γ(210). The Wulff shapes of Al and Cu are obtained according to the surface energies calculated above, respectively, as shown in Figure 3. It is obvious that Wulff shapes of Al and Cu are mainly composed of low index surfaces; the top two largest surface area of the two metals are (111) and (100) surfaces. Since both metals are in FCC structures, it is not surprising that the Wulff shapes of these two metals are very similar in terms of structure and shape. At the same time, these results are also consistent with the crystal plane groups corresponding to the XRD peaks of metallic melts in Figure 1.

**Table 1:** Theoretical values of the surface energies of various Al/Cu metal slabs.

**Surface types**	**Surface energies (J/m^**2**^)**	**Surface types**	**Surface energies (J/m^**2**^)**
Al (100)	0.96	Cu (100)	1.44
Al (110)	1.03	Cu (110)	1.53
Al (111)	0.87	Cu (111)	1.33
Al (210)	1.03	Cu (210)	1.61
Al (211)	0.96	Cu (211)	1.49
Al (221)	0.96	Cu (221)	1.47
Al (310)	1.03	Cu (310)	1.57
Al (311)	0.99	Cu (311)	1.53
Al (331)	0.99	Cu (331)	1.50
Al (511)	0.98	Cu (511)	1.52

**Figure 4:**
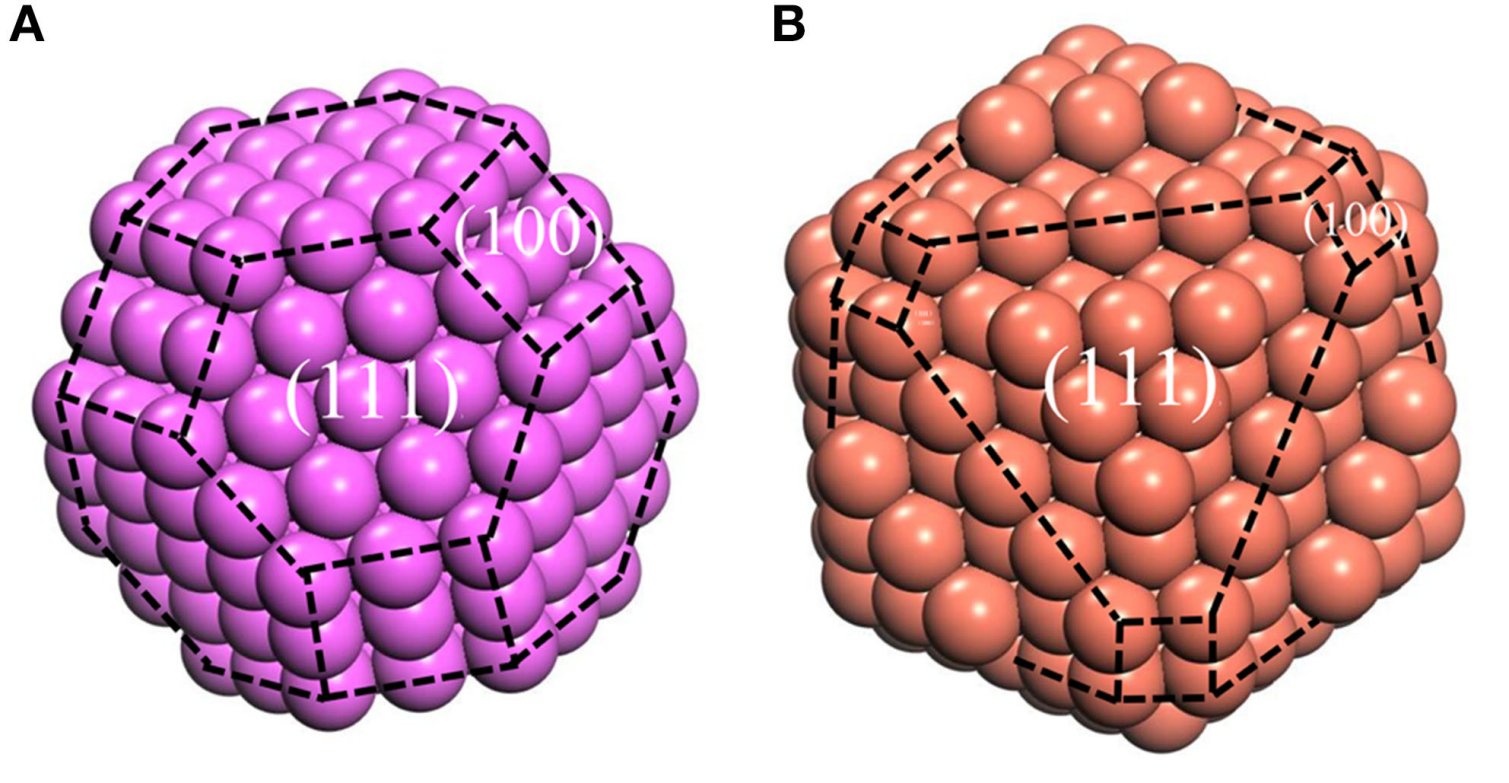
the model of atomic clusters, **(A)** Aluminum at 1,250°C, **(B)** Copper at 1,350°C. The purple and red balls represent the Al and Cu atoms, respectively. The black dotted lines represent the boundary of different surfaces.

However, the Wulff model only describes the geometric shape corresponding to the surface energies, without the definite size. Fortunately, the size can be determined by the experimental methods mentioned above. Until now, our atomic cluster models, which could be obtained based on Wulff shape, are complete, with the bulk structure inside, whose size is determined by HTXRD experiments. In this case, the atomic cluster models in Al/Cu melts can be formed easily (Figure 4). Due to the limitation of size, the complete morphology of Wulff shape cannot be reflected in an atomic cluster structure which just consists of (100) and (110) surfaces.

**Figure 5:**
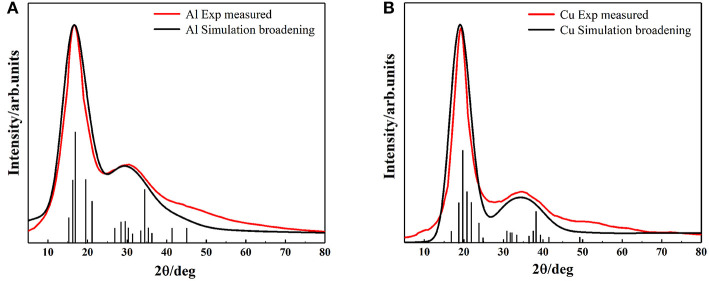
The comparison of simulation and experimental results of XRD, **(A)** Aluminum **(B)** Copper. Black vertical lines represent the diffraction patterns of our models, black and red curves represent the simulation broadening XRD and the experimental measured XRD results, respectively.

Despite the simplicity of the current model, it is capable of describing the properties of metallic melts quantitatively well. The XRD peak spectrums of Al and Cu atomic clusters were calculated by *Materials Studio* module (Figure 5). The simulation curves are broadened (using Equation 2) by multiple vertical lines (calculated XRD patterns). After the relaxation of the clusters, they hardly change their structures, but the distance of their outermost layers changes a little bit. This might be the main reason that the peaks split into several ones which are not far from each other. The simulated XRD of Wulff shape matches the XRD curve of liquid quite well, including not only the position and width of the peaks but also the relative intensity of the first and second peaks. Obviously, this model can describe the abnormal HTXRD results in Al melt (Figure 1). It can also be inferred that the Al melt is composed of Wulff atomic clusters and a large number of free atoms. So, what is the reason for the large deviation between the XRD results of Al metallic melt (composed of clusters and free atoms) and the XRD of solid ones? We believe that it is mainly caused by two reasons. First, the distortion and relaxation of atomic clusters' surfaces may lead to the splitting and deviation of the peaks in the XRD results. Second, the limited size of the clusters would definitely cause the peak to be missing in high-index surfaces. The little difference in the large angle, in our opinion, is mainly caused by: (1) the defects and deformation of the short-range orderings under such a high temperature, which is ignored in our model; and (2) our calculating the XRD patterns of cluster structures in vacuum, which means the free atoms between the clusters in melts are not considered. There will certainly be some background diffraction missing in our simulation. But if we focus on the information of peaks, not only the positions/areas of the peaks but also the relative intensity of the first and second peaks agree with the experimental data rather well. It indicates that the ordering part in the realistic melts could be well-described by our cluster model.

**Figure 6:**
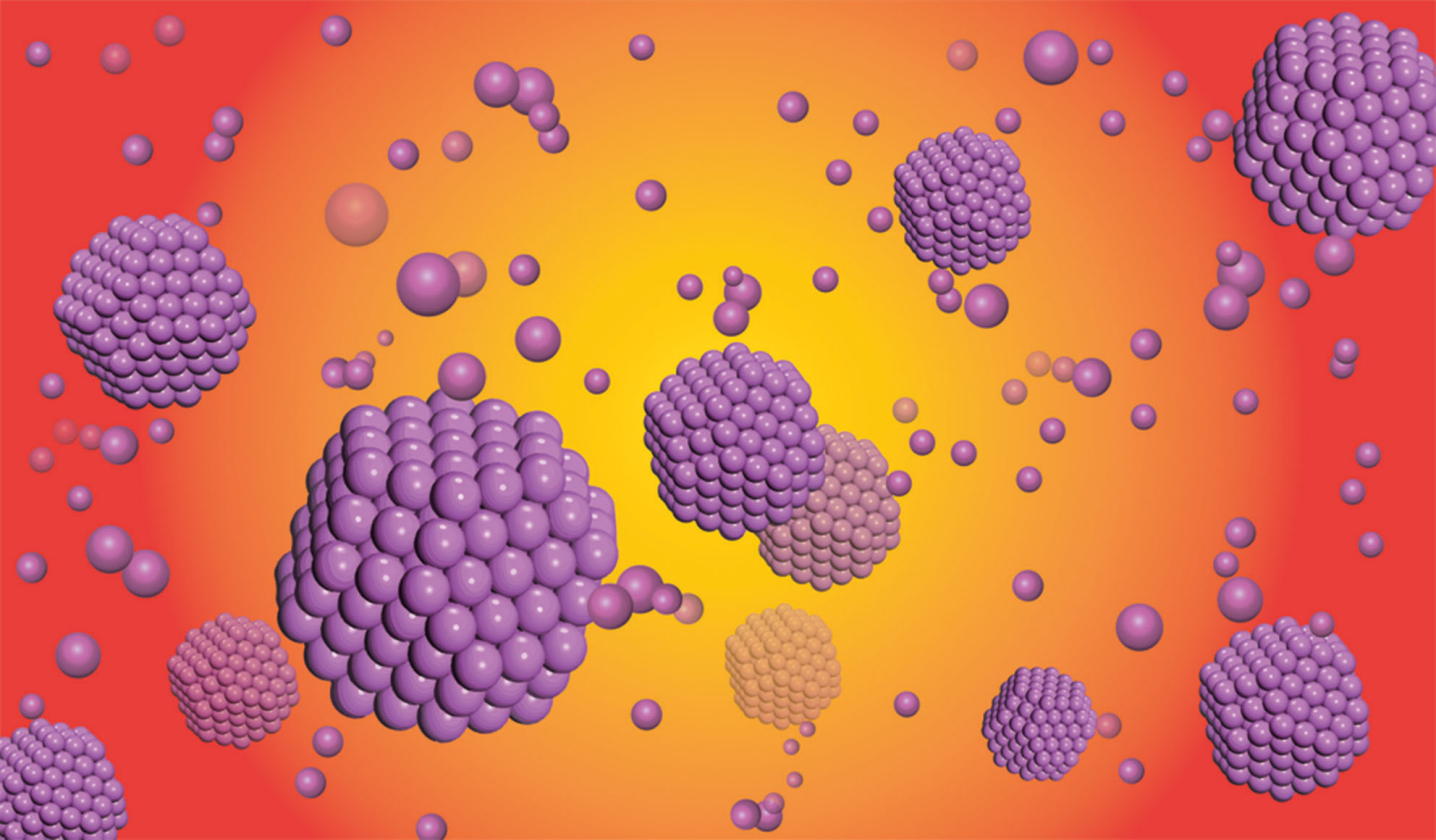
Schematic diagram of Al melt, including atomic cluster structures and free atoms.

Finally, according to our atomic cluster model, a schematic figure of Al metallic melt was given (taking Al as an example in Figure 6), and the melt structure is mainly composed of atomic cluster structures and free atoms around them. Although the single atomic cluster structures are not stable under such a high temperature, the distribution of atomic cluster structures and their number remain constant thermodynamically. This model is useful not only for the description of pure metallic melts but also for the alloys. The extension of the models to binary alloys are now in progress, with good results, and will be published thereafter.

## Conclusion

In the present work, the cluster structure model is proposed to describe the structure of metallic melts, which is determined by metal Wulff construction with the nano-sized crystal structures inside. Wulff shape is determined by surface energies of different crystal plane groups calculated by DFT, and the size is given by the PDF of the experimental XRD results of metallic melts. The validity of the current model was accessed by predicting the XRD curves of Al and Cu at high temperatures, which matched the experimental results quantitatively well, including the position and width of the peaks and the relative intensity of the first and second peaks. We hope that the model can help us to better understand the properties of metallic melts and to guide the design and fabrication of metallic structures with desired functions.

## Data Availability Statement

The raw data supporting the conclusions of this article will be made available by the authors, without undue reservation.

## Author Contributions

LS completed 90% of the work and completed the writing of manuscript. XT discussed the overall idea and research plan. YY helped to revise the manuscript. JQ talked about DFT calculation details. HL participated in the discussion on the use of Materials Studio. XL provided the main idea and paid for the research funding. All authors contributed to the article and approved the submitted version.

## Conflict of Interest

The authors declare that the research was conducted in the absence of any commercial or financial relationships that could be construed as a potential conflict of interest.
